# Morbidity of the Donor Site and Complication Rates of Breast Reconstruction with Autologous Abdominal Flaps: A Systematic Review and Meta-Analysis

**DOI:** 10.1155/2022/7857158

**Published:** 2022-06-24

**Authors:** Hatan Mortada, Taif Fawaz AlNojaidi, Razan AlRabah, Yousif Almohammadi, Raghad AlKhashan, Hattan Aljaaly

**Affiliations:** ^1^Division of Plastic Surgery, Department of Surgery, King Saud University Medical City, King Saud University and Department of Plastic Surgery and Burn Unit, King Saud Medical City, Riyadh, Saudi Arabia; ^2^Faculty of Medicine, Imam Mohammad Ibn Saud University, Riyadh, Saudi Arabia; ^3^Faculty of Medicine, King Saud University, Riyadh, Saudi Arabia; ^4^Faculty of Medicine, Alfaisal University, Riyadh, Saudi Arabia; ^5^Division of Plastic Surgery, Department of Surgery, King Abdulaziz University Hospital, Jeddah, Saudi Arabia

## Abstract

**Background:**

Numerous studies have evaluated the use of autologous abdominal tissue for breast reconstruction; nevertheless, complications and donor site morbidity rates vary significantly. The study aims to compare the literature regarding morbidity of the donor site and complication rates of breast reconstruction with autologous abdominal flaps.

**Methods:**

The databases of MEDLINE, EBSCO, Scopus, Wiley Library, and Web of Sciences were searched for studies that compared different flaps in terms of complications and donor site morbidity. The procedures studied included pedicled transverse rectus abdominis myocutaneous ﬂap (pTRAM), free TRAM (fTRAM), deep inferior epigastric perforator (DIEP), and superﬁcial inferior epigastric artery perforator (SIEA) flaps. A total of 34 studies were included. Of these, 28 were retrospective studies and 9 were prospective cohort studies.

**Results:**

When compared to DIEP, fTRAM flaps were found to have a decreased incidence of flap fat necrosis, hematoma, and total thrombotic events, yet a higher risk of donor site hernia/bulging. pTRAM flaps were also associated with an increased risk of hernia/bulging at the donor site, as well as wound infection, yet flap hematoma was less common. On the other hand, SIEA flaps showed the lowest risk of donor site hernia/bulging while still having a high risk of wound infection.

**Conclusion:**

fTRAM procedures comparatively had the least complications. However, regarding flap choice, patients would benefit most from a case-by-case analysis, taking into consideration individual risk factors and preferences.

## 1. Introduction

Breast cancer is the most common cancer among women. According to the World Health Organization, in 2020, there were 2.3 million women diagnosed with breast cancer [1]. After mastectomy, breast reconstruction surgery is considered a crucial step toward more comprehensive breast cancer treatment and management [2]. For women undergoing breast reconstruction surgery, two main points are considered: timing and type of reconstruction. Breast reconstruction can be done either at the time of mastectomy *(immediate reconstruction)* or later *(delayed reconstruction).* Based on the type of procedure, breast reconstruction can be classified into implant-based reconstruction (IBR) or autologous reconstruction (AR) [3]. Throughout the years, multiple donor sites and options have been described for AR. However, abdominal-based flaps continue to be the mainstay for AR [4]. In 1982, Hartrampf et al. described the transverse rectus abdominis myocutaneous (TRAM) flap, which provides a soft, ptotic, and aesthetically pleasing reconstruction similar to the natural breast [5]. However, the abdominal wall integrity is compromised due to the use of rectus muscle and fascia, resulting in abdominal bulge and hernia, which is recognized as a limitation of the TRAM flap [6]. To reduce abdominal donor site morbidity by harvesting less muscle, the TRAM flap has undergone numerous modifications, resulting in different techniques such as muscle-sparing TRAM (MS-TRAM), deep inferior epigastric perforator (DIEP), and superficial inferior epigastric artery (SIEA) flaps [7–9]. Sailon et al. conducted a systematic review in 2009 to determine the difference in patient selection, flap-related complications, and donor site morbidity. They found a statistically significant difference between DIEP and MS-TRAM flaps in fat necrosis and total necrosis, but not partial necrosis rates, whereas for donor site morbidity, there was no statistically significant difference. Also, the study interprets the lack of a statistically significant difference due to the insufficient sample size, resulting in low statistical power and type II error [10]. Egeberg et al. conducted a meta-analysis in 2012 to compare the donor site morbidities of SIEA, DIEP, and MS-TRAM flaps and found a 20% reduced risk of bulging when DIEP flaps were used compared to MS-TRAM flaps. However, there was no statistically significant difference. Also, a limitation in the included studies was the small sample size [11]. Another study found no statistically significant difference in flap-related complications (vessel thrombosis, partial or total flap loss, and fat necrosis) or donor site morbidity (abdominal bulge) between the MS-TRAM and DIEP flaps. The study concluded that determining flap superiority is challenging due to different harvesting techniques between surgeons and different measures used to evaluate the donor site morbidity. Thus, higher-quality studies are needed to compare different types of flaps and determine the flap choice for each patient [12]. Therefore, this systematic review and meta-analysis aim to review and compare the literature regarding the morbidity of the donor site and the complication rates of breast reconstruction with autologous abdominal flaps.

## 2. Materials and Methods

### 2.1. Literature Search

For this study, we followed the International Prospective Register of Systematic Reviews (PROSPERO) statement (ID: CRD42021281876) [13]. A systematic search of MEDLINE, EBSCO, Wiley Library, Scopus, and Web of Science databases was conducted in September 2021. The databases were screened by Rayyan (https://www.rayyan.ai/) [14]. We used the keywords TRAM, pTRAM, fTRAM, transverse rectus abdominis myocutaneous, DIEP, deep inferior epigastric perforator, SIEA, superficial inferior epigastric artery perforator, post-operative complications, complications, outcome, and donor site morbidity or morbidity.

### 2.2. Study Selection

English studies that assessed the complications following breast reconstruction with autologous abdominal flaps were included in this systematic review. Studies met the inclusion criteria if they had more than ten patients and compared flap complication outcomes, or donor site morbidities and complications for pedicled transverse rectus abdominis myocutaneous flaps (pTRAM), free TRAM (fTRAM), deep inferior epigastric perforator (DIEP), and superficial inferior epigastric artery perforator (SIEA) flap in adults 18 years or older. Review articles, meta-analysis/systematic reviews, case reports, economic analyses, animal studies, cadaver studies, narrative reviews, and editorials were excluded. Studies that exclusively evaluated only one type of autologous abdominal flap or reported no outcome of interest were also excluded. Articles were retrieved and screened by four independent investigators, and a fifth investigator then resolved any disagreements.

### 2.3. Data Extraction

Full-text articles were retrieved and screened. A single investigator retrieved data from each report. General information was collected from the articles, including author, country, design, sample size, age of patients, and year of publication. The target outcomes of the incidence of donor site complications, rates of each type of complication, and risk factors for complications were extracted. In addition, implant complications were also extracted.

### 2.4. Statistical Analysis

Statistical analysis was conducted using R software (RStudio 2021.09.0 Build 351 for Windows). Generally, the analysis consists of two approaches. First, we explored the single proportions of all the flap and donor site complications in each intervention arm using the *metaprop* package in R. The overall proportions of complications were transformed using logit transformation in a random-effects environment. Statistical differences in the proportions were assessed using a *Q* test to test for group-based differences. Second, a network meta-analysis of the primary outcomes was carried out using the netmeta package [15]. A random-effects network meta-analysis was employed using the frequentist approach [16]. The network approach was utilized to combine data from studies with two or more arms and to provide reliable direct and indirect comparisons of the treatment effects. This would optimize the estimated precision and provide an insight into the relative ranking of the risk of complications while accounting for the association between effect sizes [17]. Effect sizes were expressed as the relative risk (RR) and the respective 95% confidence interval (95% CI) to determine the ratio between the incidences of a complication in a given surgical arm versus the control arm. In the present network model, the DIEP arm was considered the common comparator (control arm). The meta-analysis was not performed for the complications which are reported in ≤3 studies. Heterogeneity testing consisted of a within-design heterogeneity assessment using an *I*^2^ test and a between-study inconsistency analysis. *Inconsistency* was defined as the variation between direct and indirect evidence. It was assessed locally using net splitting and globally by constructing a full design-by-treatment interaction random-effects model [18]. League tables were produced to express all the potential pairwise comparisons of surgical complications in off-diagonal cells [19]. Furthermore, the effect estimates of the risk of complications were visually assessed by producing network forest plots. The risk of complications was also ranked using P scores (range 0–1), where higher ranks (close to 1) indicated that the surgical technique was associated with a lower risk of complications. Assessment of publication bias was performed via producing comparison-adjusted funnel plots and was corroborated by Egger's test. A *pvalue* of <0.05 indicated statistical significance [20].

## 3. Results

### 3.1. Literature Findings

**Figure 1:**
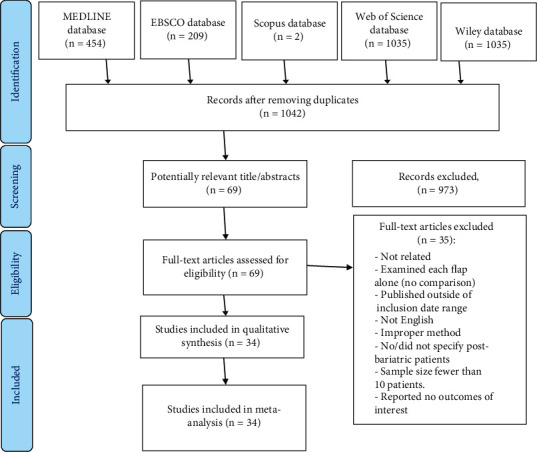
PRSIMA diagram for the systematic review.

A total of 2106 publications were found after a thorough search, including 454 from MEDLINE databases, 209 from EBSCO, 2 from Scopus, 1035 from Web of Science, and 406 articles from Wiley Library. For this systematic review, we initially retrieved 69 full-text publications. However, after implementing the exclusion criteria, only 34 articles between January 2000 and November 2021 were included (Figure 1). Out of the included studies, 5 were prospective studies [21–25] and 29 were retrospective chart reviews [4, 9, 12, 26–51]. None of the selected studies were randomized controlled trials. Studies included data of 60551 patients, of whom 20052 (33.12%) underwent fTRAM, 10298 (17.01%) underwent pTRAM, 29259 (48.32%) underwent DIEP, and 942 (1.56%) underwent SIEA surgeries. More details about the characteristics of studies and patients are demonstrated in Table 1.

### 3.2. Network Structure

**Figure 2:**
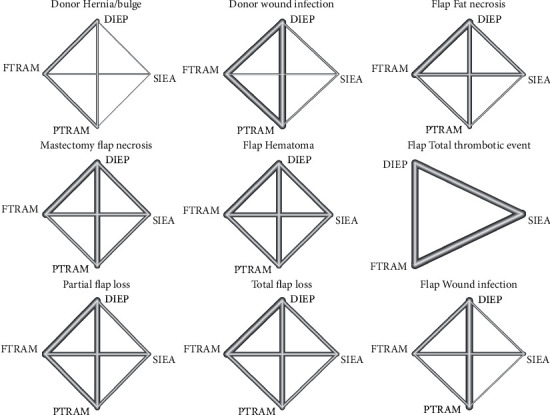
Network maps of eligible pairwise comparisons for the risk of complications across different autologous breast reconstruction techniques. The thickness of lines indicates the number of included studies in each comparison.

The establishment of the network was not possible for distinct complications which comprised 2 intervention arms only (flap intraoperative arterial thrombosis and intraoperative venous thrombosis). Additionally, the following complications were excluded because they were reported in ≤3 studies: fat necrosis, hematoma, seroma, abdominal fat necrosis, and umbilical necrosis at the donor site, as well as seroma, abdominal flap necrosis, and post-operative arterial/venous thrombosis at the flap site. The network structures of the remaining outcomes are illustrated in Figure 2, and the numbers of patients in each comparison are listed in Table 2. All the networks comprised direct comparisons among different surgical techniques except donor hernia/bulge (no direct pairwise comparisons between pTRAM and SIEA) and flap total thrombotic events (the outcome was not investigated in pTRAM procedures, Figure 2).

### 3.3. Testing for Heterogeneity and Inconsistency

The analysis of within-design heterogeneity, which reflects the variation in true effect sizes between studies with the same design, indicated a statistically significant heterogeneity in the effects sizes of flap fat necrosis (*Q* = 88.07, *p* < 0.0001), flap hematoma (*Q* = 47.49, *p* < 0.0001), and partial flap loss (*Q* = 43.94, *p* < 0.0001). On the other hand, the variation between designs (inconsistency) was significant in four outcomes, including flap hematoma (*Q* = 35.21, *p* < 0.0001), flap total thrombotic events (*Q* = 15.32, *p*), and partial (*Q* = 14.85, *p*=0.005) and total flap loss (*Q* = 12.70, *p*=0.048). These findings supported the application of random-effects models in the network analysis (Table 2).

### 3.4. The Risks of Morbidity of the Donor Site and Flap Complications

**Figure 3:**
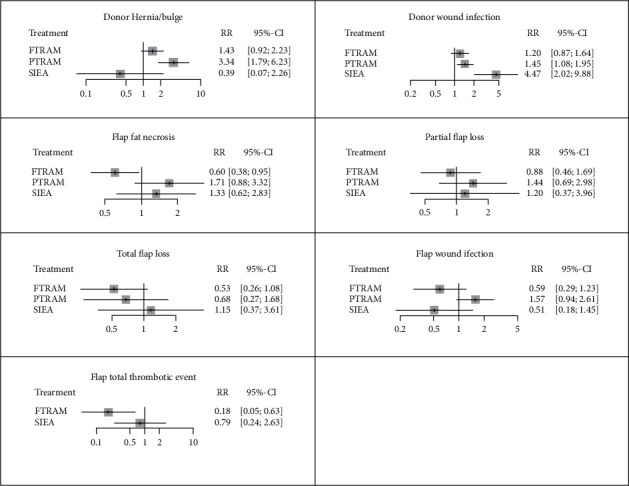
Forest plots depicting the relative risks (RRs) of donor site morbidity and flap-related complications after breast reconstruction surgeries.

The outcomes of the risk of morbidity at the donor site and flap complications are depicted in Figure 3. Considering DEIP procedures as a reference intervention, fTRAM flaps reduced the risks of flap fat necrosis (RR = 0.60, 95% CI, 0.38 to 0.95), hematoma (RR = 0.54, 95% CI, 0.32 to 0.89), and total thrombotic events (RR = 0.18, 95% CI, 0.05 to 0.63); nevertheless, they were associated with higher risks of hernia/bulge (RR = 1.43, 95% CI, 0.92 to 2.23).

pTRAM flaps were associated with significantly higher risks of hernia/bulge (RR = 3.34, 95% CI, 1.79 to 6.23) and wound infection (RR = 1.45, 95% CI, 1.08 to 1.95) at the donor site. However, the risk of flap hematoma was lower after pTRM flap (RR = 0.52, 95% CI, 0.29 to 0.93). Finally, SIEA flap increased the risk of wound infection compared to DIEP (RR = 4.47, 95% CI, 2.02 to 9.88). More details about the risks of morbidity/complications after different surgeries relative to each other are provided in the net league tables (Table 3).

### 3.5. Risk Ranking

The P score analysis was implemented in the present study to quantify the certainty that a given intervention is better than another intervention, averaged over all other interventions. This type of analysis has been used as an alternative to SUCRA scoring which has been used in the Bayesian network models [52]. Based on P scores, results indicated that the highest ranks of reducing the risk of hernia/bulge and wound infection at the donor sites were apparent after SIEA and DIEP surgeries, respectively (Table 4). Regarding flap complications, fTRAM surgeries ranked the best in terms of reducing the risk of five complications, including fat necrosis, mastectomy flap necrosis, total thrombotic events, and partial and total flap loss. In addition, SIEA procedures ranked first in reducing the risk of flap wound infection and hernia/bulge (Table 5).

### 3.6. Publication Bias

**Figure 4:**
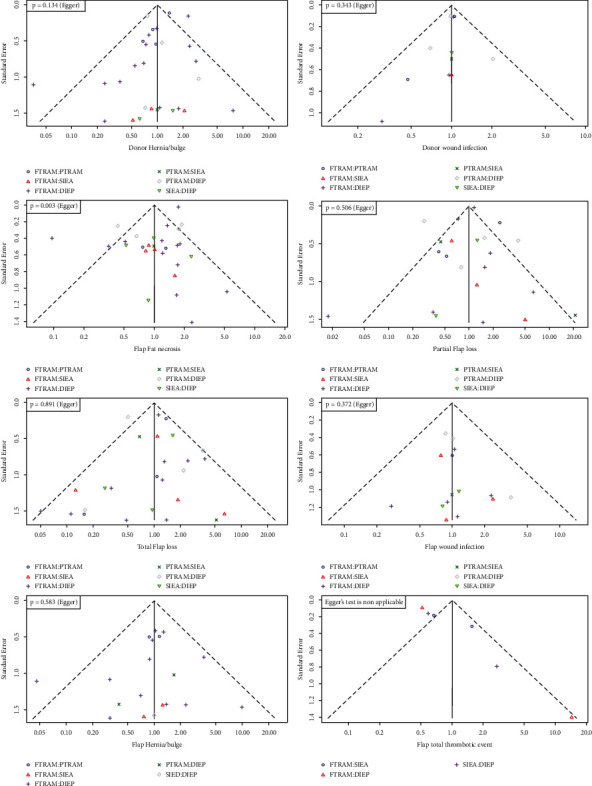
Funnel plots depicting the risk of publication bias in eligible pairwise comparisons of the network.

Visual assessment of funnel plots indicated that the eligible pairwise comparisons were asymmetrically distributed around the effect estimate, where small-sized studies (with higher standard errors) were more likely to be published when they reported high risks of complications at the donor site (hernia/bulge and wound infection, Figure 4). However, the results of Egger's test indicated a lack of statistically significant publication bias (*p*=0.088 for hernia/bulge and *p*=0.343 for wound infection). There was no risk of publication bias for the complications of the flap except fat necrosis, where small-sized studies were not published if they reported low risks of fat necrosis (Figure 4). This was corroborated by the results of Egger's test (*t* = −3.31, *p* value = 0.003).

## 4. Discussion

Microsurgical breast reconstruction with abdominal-based flaps is a challenging and complex procedure and an excellent choice for patients who want to avoid implants. However, it has a high risk of wound and surgical complications at the donor and recipient sites [53]. Hence, the literature has reported no statistically significant differences between abdominal-based breast reconstruction flap-related complications or donor site morbidity [12]. As a result, higher-quality research is required to evaluate the different choices of flaps for each patient. Thus, we have conducted this systematic review and meta-analysis to examine and evaluate the literature on donor site morbidity and complication rates in breast reconstruction using different autologous abdominal flap techniques, including TRAM, pTRAM, fTRAM, DIEP, and SIEA. Our study includes data from 60551 patients, 20052 (33.12%) of whom have undergone fTRAM procedure, 10298 (17.01%) pTRAM, 295259 (48.32%) DIEP, and 942 (1.56%) SIEA flaps. This is in agreement with the literature that the most popular and reliable free flaps are fTRAM and DIEP [54]. We found a tendency toward a higher likelihood of abdominal bulge/hernia after pTPAM compared to DIEP flaps, which supports the literature that losing muscle makes the fascial abdomen vulnerable to weakness [11, 55]. It is well evident in the literature that following pTRAM, vascular-related complications (e.g., flap loss and fat necrosis) are more likely to occur due to the tunneling of a pedicled flap up the chest, causing blood flow restriction by kinking or compression of the superior epigastric arteries, or that the pedicle blood supply offers decreased perfusion [34, 36, 56].

Nevertheless, in our study, we found that pTRAM increased the risk of hernia/bulge (RR = 3.34, 95% CI, 1.79 to 6.23) and wound infection (RR = 1.45, 95% CI, 1.08 to 1.95) at the donor site. On the other hand, DIEP flaps benefit from the improved blood supply provided by the inferior epigastric veins and the flow dynamics provided by the internal mammary or thoracodorsal systems. Although free abdominally based flaps have a lower risk of complications, pTRAM flaps may still be appropriate for certain patients, especially when microsurgery is not an option due to surgeon skill, patient comfort, or patient desires based on risk-weighted judgments.

In this study, fTRAM has a much higher incidence of abdominal bulge/hernia than other types of autologous abdominal breast reconstruction. This is consistent with the literature since obese patients, in comparison with nonobese, had a higher incidence of abdominal bulge/hernia with fTRAM flaps than with DIEP flaps [57]. As a result, if feasible, other techniques (e.g., DIEP flaps) need to be considered in this group of patients. Due to greater tension being placed on the weaker abdominal fascia, obesity may worsen and increase the likelihood of abdominal bulge/hernia after fTRAM flaps [32]. Nevertheless, fat necrosis, mastectomy flap necrosis, total thrombotic events, and partial and total flap loss were the least common complications associated with fTRAM surgeries. There are no established criteria to aid in determining which vessels will provide adequate tissue perfusion; thus, it is recommended to conduct a high-quality study to determine which imaging techniques are best to assess the vessels' flow and anatomy to reduce the incidence of these complications.

Regarding flap fat necrosis, the risk of publication bias was considerable (both visually and statistically). For hernia/bulge and wound infection at the donor site, the risk of publication bias was visually obvious (although not statistically significant). An essential part of our statistical analysis is assuming that patients undergoing abdominal autologous reconstruction are equivalent candidates. There was no selection bias in choosing which flap to use. Therefore, to reduce the magnitude of this assumption, studies that exclusively evaluated only one type of autologous abdominal flap were excluded, and only studies that evaluated the different types of flaps concurrently were included. Another potential source of bias is the lack of standardization of the surgical techniques due to the surgeon's particular variation of each flap procedure.

The main strength of this study is that it compares the four most common types of abdominal autologous flaps with donor site morbidity and complication rates in breast reconstruction. Most studies mentioned in the literature compared two flaps only. Thus, a future systematic review and meta-analysis are needed to evaluate the quality of the published data and compare them to each other to determine the appropriate flap choice for each patient.

Furthermore, although this study did not pay attention to the nonclinical related variables to the patient, such as preoperative physical activity, it is crucial to note that the patient's expectations and objectives are essential in selecting the appropriate flap. Due to a lack of extractable data, we could not adjust for any preoperative risk variables. More high-quality prospective cohort trials are required to estimate how other patient-related variables can impact the complication rates and overall patient satisfaction. Another drawback in our systematic review and meta-analysis is the lack of randomized control trials, owing to ethical considerations. Despite that, we recommend conducting a high-quality randomized control trial that will be able to address the biases that were present in the published studies. Another limitation of our study is that the aesthetic results for the breast and the patient's satisfaction were not compared, which is an essential factor in the consideration of flap choice due to the insufficient data and standardized tools used to assess those outcomes in the literature.

## 5. Conclusion

The use of the abdominal wall for autologous breast reconstruction is an optimized procedure for both aesthetic and functional outcomes. Our findings demonstrate that the morbidity of the donor site and complications following abdominally based breast reconstruction techniques depend on the flap type and some patient-related factors. When possible, fTRAM reconstructions should be performed as they have a lower risk of complications than any other flap type compared in this study. However, using SIEA in obese patients may reduce hernia/bulge donor site risk. Therefore, selecting an autologous abdominal flap is a multifactorial decision that should be patient-oriented rather than procedure-oriented to minimize donor site morbidity and complications.

## Figures and Tables

**Table 1:** Characteristics of the included studies.

^*#*^	Author and year	Country	Study design	Study arms			
				fTRAM	pTRAM	DIEP	SIEA
1	Selber et al. 2008 [26]	USA	Retrospective cohort study	569	NA	NA	69
2	Momoh et al. 2012 [4]	USA	Retrospective cohort study	NA	197	217	NA
3	Nahabedian et al. 2005 [23]	USA	Retrospective cohort study	113	NA	110	NA
4	Nahabedian et al. 2002 [27]	USA	Retrospective cohort study	143	NA	20	NA
5	Chevray. 2004 [24]	USA	Prospective cohort study	25	NA	8	14
6	Bajaj et al. 2006 [12]	USA	Retrospective cohort	155	NA	48	NA
7	Chun et al. 2010 [29]	USA	Retrospective cohort	105	NA	85	NA
8	Schaverien et al. 2007 [30]	UK	Retrospective cohort	30	NA	30	NA
9	Erdmann-Sager et al. 2018 [21]	USA	Prospective cohort	115	89	445	71
10	Scheer et al. 2006 [31]	Canada	Retrospective cohort	46	NA	84	NA
11	Chang et al. 2016 [32]	USA	Retrospective cohort	340	NA	573	NA
12	Nahabedian et al. 2002 [9]	USA	Retrospective cohort	108	37	10	NA
13	Langer et al. 2010 [33]	USA	Retrospective cohort	254	NA	451	1
14	Garvey et al. 2006 [34]	USA and Canada	Retrospective cohort	NA	94	96	NA
15	Agarwal. Gottlieb. 2007 [35]	USA	Retrospective cohort	8	NA	6	NA
16	Kroll. 2000 [36]	USA	Retrospective cohort	279	NA	31	NA
17	Holoyda et al. 2019 [37]	USA	Retrospective cross-sectional study	3007	2180	8007	154
18	Chen et al. 2007 [22]	USA	Prospective cohort	159	NA	41	NA
19	Kwok et al. 2019 [38]	USA	Retrospective cross-sectional study	4461	5079	6206	245
20	Andree et al. 2008 [39]	Germany	Retrospective cohort	148	NA	201	NA
21	Vega et al. 2006 [40]	USA	Retrospective cohort	11	NA	2	10
22	Wan et al. 2010 [41]	USA	Retrospective cohort	57	NA	200	NA
23	Andrades et al. 2008 [42]	USA	Retrospective cohort	154	147	NA	NA
24	Seidenstuecker et al. 2011 [23]	Germany	Prospective cohort	224	NA	400	NA
25	Takeishi et al. 2008 [43]	Japan	Retrospective study	79	NA	30	NA
26	Baumann et al. 2010 [25]	USA	Prospective study	120	NA	71	37
27	Masoomi et al. 2014 [44]	USA	Retrospective cohort	6556	NA	8153	304
28	Wu et al. 2014 [45]	USA	Retrospective cross-sectional study	NA	69	69	NA
29	Futter et al. 2000 [46]	Scotland	Retrospective study	27	NA	23	NA
30	Vyas et al. 2008 [47]	USA	Retrospective review	37	NA	128	NA
31	Coroneos et al. 2015 [48]	Canada	Retrospective cohort study	NA	NA	75	37
32	Shubinets et al. 2016 [49]	USA	Retrospective cohort study	2474	2406	3366	NA
33	Bonde et al. 2006 [50]	Denmark	Retrospective cohort study	4	NA	25	NA
34	Zhong et al. 2014 [51]	Canada	Retrospective study	244	NA	48	NA

**Table 2:** Descriptive data of the number of complications (events) and total number of patients as well as the outcomes of heterogeneity and inconsistency analyses.

Outcome	*k* ^*∗*^	Events/total				Within designs (heterogeneity)		Between designs (inconsistency)		Consistency^¥^	
		DIEP	FTRAM	PTRAM	SEIA	*Q*	*p*	*Q*	*p*	*Q*	*p*
Donor hernia/bulge	18	115/4726	247/4554	218/2773	0/154	17.01	0.149	24.19	**0.002**	41.20	**0.004**
Donor wound infection	4	191/3991	155/2635	188/2589	9/71	NA	NA	4.43	0.351	4.43	0.351
Flap fat necrosis	19	253/1679	414/2454	137/596	25/228	88.07	**<0.0001**	11.94	0.102	1.60	0.979
Mastectomy flap necrosis	7	47/663	53/1107	15/158	7/140	2.05	0.562	1.52	0.678	1.52	0.678
Flap hematoma	13	624/15452	250/9008	150/7639	45/549	47.49	**<0.0001**	35.21	**<0.0001**	12.51	**0.028**
Flap total thrombotic event	4	2124/8312	146/7171	NA	50/410	NA	NA	15.32	**0.002**	15.32	**0.002**
Partial flap loss	12	166/7390	351/6330	68/5581	6/385	43.94	**<0.0001**	14.85	**0.005**	0.22	0.994
Total flap loss	14	133/7493	63/6310	44/5606	8/395	15.37	0.119	12.70	**0.048**	6.60	0.359
Flap wound infection	8	41/891	33/908	28/380	5/150	1.88	0.598	2.48	0.779	2.48	0.778

^*∗*^The number of studies in each comparison; ^*¥*^consistency under the assumption of a full design-by-treatment interaction random-effects model.

**Table 3:** The relative risks of donor site morbidity and flap complications across different breast reconstruction techniques.

*Donor hernia/bulge*				*Donor wound infection*			
FTRAM				FTRAM			
0.43 (0.24–0.77)	PTRAM			0.82 (0.60–1.13)	PTRAM		
3.66 (0.65–20.74)	8.53 (1.42–51.32)	SIEA		0.27 (0.12–0.61)	0.33 (0.15–0.73)	SIEA	
1.43 (0.92–2.23)	3.34 (1.79–6.23)	0.39 (0.07–2.26)	DIEP	1.20 (0.87–1.64)	1.45 (1.08–1.95)	4.47 (2.02–9.88)	DIEP

*Flap fat necrosis*				*Mastectomy flap necrosis*			
FTRAM				FTRAM			
0.35 (0.17–0.74)	PTRAM			0.77 (0.35–1.70)	PTRAM		
0.45 (0.21–0.97)	1.29 (0.50–3.30)	SIEA		0.82 (0.35–1.93)	1.07 (0.41–2.74)	SIEA	
0.60 (0.38–0.95)	1.71 (0.88–3.32)	1.33 (0.62–2.83)	DIEP	0.90 (0.53–1.53)	1.17 (0.62–2.21)	1.10 (0.50–2.38)	DIEP

*Partial flap loss*				*Total flap loss*			
FTRAM				FTRAM			
0.61 (0.28–1.35)	PTRAM			0.78 (0.29–2.05)	PTRAM		
0.73 (0.23–2.36)	1.19 (0.35–4.09)	SIEA		0.46 (0.15–1.41)	0.59 (0.16–2.10)	SIEA	
0.88 (0.46–1.69)	1.44 (0.69–2.98)	1.20 (0.37–3.96)	DIEP	0.53 (0.26–1.08)	0.68 (0.27–1.68)	1.15 (0.37–3.61)	DIEP

*Flap hematoma*				*Flap total thrombotic event*			
FTRAM				FTRAM			
1.04 (0.54–1.99)	PTRAM			0.23 (0.07–0.68)	SIEA		
0.37 (0.19–0.72)	0.36 (0.17–0.74)	SIEA		0.18 (0.05–0.63)	0.79 (0.24–2.63)	DIEP	
0.54 (0.32–0.89)	0.52 (0.29–0.93)	1.45 (0.76–2.75)	DIEP				

*Flap wound infection*							
FTRAM							
0.38 (0.16–0.88)	PTRAM						
1.17 (0.45–3.02)	3.10 (0.99–9.65)	SIEA					
0.59 (0.29–1.23)	1.57 (0.94–2.61)	0.51 (0.18–1.45)	DIEP				

Data are expressed as relative risks (95% confidence intervals). The outcomes can be interpreted from the left to right direction to indicate low/high risks.

**Table 4:** P score-based ranking of the risks of donor site morbidity after four breast reconstruction techniques.

*Hernia/bulge*	
SIEA	0.924
DIEP	0.697
FTRAM	0.375
PTRAM	0.004
*Wound infection*	
DIEP	0.954
FTRAM	0.671
PTRAM	0.374
SIEA	0.001

**Table 5:** P score-based ranking of the risks of flap complications after four breast reconstruction techniques.

*Flap fat necrosis*	
FTRAM	0.987
DIEP	0.576
SIEA	0.317
PTRAM	0.120
*Mastectomy flap necrosis*	
FTRAM	0.688
DIEP	0.542
SIEA	0.430
PTRAM	0.341
*Flap hematoma*	
PTRAM	0.842
FTRAM	0.816
DIEP	0.298
SIEA	0.045
*Total thrombotic event*	
FTRAM	0.996
SIEA	0.326
DIEP	0.178
*Partial flap loss*	
FTRAM	0.745
DIEP	0.602
SIEA	0.431
PTRAM	0.222
*Total flap loss*	
FTRAM	0.857
PTRAM	0.632
DIEP	0.279
SIEA	0.232
*Flap wound infection*	
SIEA	0.833
FTRAM	0.760
DIEP	0.380
PTRAM	0.027

## Data Availability

Data are available on request to the corresponding author.
